# Targeting Ventricular Arrhythmias in Non-Ischemic Patients: Advances in Diagnosis and Treatment

**DOI:** 10.3390/diagnostics15040420

**Published:** 2025-02-09

**Authors:** Laura Adina Stanciulescu, Maria Dorobantu, Radu Vatasescu

**Affiliations:** 1 Department of Cardiothoracic Pathology, Faculty of Medicine, Carol Davila University of Medicine and Pharmacy Bucharest, 050474 Bucharest, Romania; laura-adina.stanciulescu@drd.umfcd.ro (L.A.S.); radu_vatasescu@yahoo.com (R.V.); 2 Cardiology Department, Clinical Emergency Hospital Bucharest, 014461 Bucharest, Romania; 3 Romanian Academy, 010071 Bucharest, Romania

**Keywords:** ventricular arrhythmias, non-ischemic cardiomyopathy, catheter ablation, epicardial ablation, bipolar ablation, stereotactic body radiation therapy, substrate ablation, multimodality cardiovascular imaging

## Abstract

Ventricular arrhythmias (VAs) in non-ischemic cardiomyopathy (NICM) present significant clinical challenges due to their diverse etiologies and complex arrhythmogenic substrates, which differ from those in ischemic heart disease. Recent advancements in imaging, electrophysiological mapping, and ablative therapy have improved the management of these arrhythmias. This review examines the spectrum of NICM subtypes, discussing their pathophysiology, prevalence, genetic determinants, and associated arrhythmias. It also explores contemporary ablative techniques, including epicardial, bipolar, and irrigated approaches, as well as emerging modalities such as stereotactic body radiation therapy (SBRT). The role of novel technologies, including high-resolution mapping and artificial intelligence, is considered in refining diagnosis and treatment. This article provides a comprehensive overview of current management strategies and discusses future directions in the treatment of VAs in NICM patients.

## 1. Introduction

Ventricular arrhythmias (VAs) are a leading cause of morbidity and mortality in patients with non-ischemic cardiomyopathy (NICM), posing significant challenges due to the heterogeneity of this condition. NICM encompasses a diverse group of myocardial disorders unrelated to coronary artery disease, each with unique structural and electrophysiological characteristics that influence arrhythmogenesis. This diversity requires a tailored approach to diagnosis and treatment, guided by a comprehensive understanding of the underlying substrates and mechanisms.

Unlike ischemic cardiomyopathy, where mostly endocardial scar-related macro-re-entry predominates, VAs in NICM often involve diffuse fibrosis, mid-myocardial or epicardial substrates, and heterogeneous electrical remodeling, making management particularly complex and contributing to a more guarded prognosis for these patients. Recent advancements in ablation techniques have shown promise in addressing these challenges and improving outcomes, although their optimal application requires further refinement. Continued research is essential to better characterize arrhythmogenic mechanisms and develop targeted strategies to reduce the burden of VAs in this patient population.

## 2. Defining Non-Ischemic Cardiomyopathies

NICMs are myocardial disorders unrelated to coronary artery disease. They include several subtypes, each with distinct definitions, pathophysiology, prevalence, genetic determinants, and arrhythmic profiles. It is hard to appreciate the overall prevalence of NICM within the general population; however, despite being an umbrella term used to encompass a large variety of phenotypes, it remains less common than ischemic heart disease (IHD).

### 2.1. Dilated Cardiomyopathy (DCM)

Traditionally, DCM has been characterized by left ventricular (LV) enlargement and impaired systolic function, occurring in the absence of abnormal loading conditions or coronary artery disease. However, the DCM phenotype represents a broad classification encompassing various genetic and acquired causes, which can influence both the VT substrate and the outcomes of ablation procedures. It is the most common form of NICM, with a prevalence of approximately 1 in 250 individuals. DCM can be idiopathic or familial, with up to 50% of cases linked to genetic mutations, particularly in genes encoding cytoskeletal and sarcomeric proteins. The pathophysiology involves myocardial fiber degeneration, leading to ventricular dilation and impaired contractility, which leads to myocardial fibrosis and electric remodeling. The characteristics of the substrate, including common scar areas and usual VT exit points are determined by the specific etiology and can exhibit significant variability depending on the nature of the arrhythmogenic substrate. The fibrosis distribution in DCM, however, usually follows one of the two most common patterns: either anteroseptal or involving the base of the LV lateral wall [1].

#### 2.1.1. SCN5A Mutations

A subtype of DCM is associated with SCN5A mutations, which frequently result in a dilative phenotype. Mutations in the SCN5A gene, encoding the cardiac sodium channel Nav1.5, can lead to a spectrum of cardiac disorders, including Brugada syndrome and progressive cardiac conduction disease. These mutations disrupt sodium channel function, leading to altered cardiac excitability and conduction. Patients may present with various arrhythmias, including VT and atrial fibrillation (AF), and are at increased risk for sudden cardiac death (SCD). Arrhythmogenic substrates are frequently localized to the RV outflow tract or regions with conduction abnormalities [2].

#### 2.1.2. Laminopathies

Mutations in the LMNA gene, encoding nuclear envelope proteins lamin A and C, cause a form of familial dilated cardiomyopathy. These mutations lead to structural and functional abnormalities in the nuclear envelope, resulting in myocardial degeneration and fibrosis. Laminopathies are associated with a high incidence of arrhythmias, including AF, atrioventricular block, and VT, often necessitating early consideration for device therapy due to the risk of SCD. The arrhythmogenic substrate typically localizes to the septum and basal LV anterior wall, but also the basal inferior wall and the mitroaortic continuity. Ablative treatment in these patients is usually linked to suboptimal outcomes, including progression to end-stage heart failure, a high incidence of VT recurrence and an overall higher mortality [3,4].

#### 2.1.3. Arrhythmogenic Right Ventricular Cardiomyopathy (ARVC) with LV Involvement

Although ARVC is recognized as a distinct subtype of cardiomyopathy, cases involving LV structural changes are frequently classified within the DCM spectrum. The classification arises from their common presentation with a dilative phenotype, driven by fibrofatty myocardial replacement, which oftentimes leads to progressive LV systolic dysfunction and eventual LV enlargement.

### 2.2. Hypertrophic Cardiomyopathy (HCM)

Defined by unexplained left ventricular hypertrophy, HCM has a prevalence of about 1 in 500. It is predominantly inherited in an autosomal-dominant pattern, with mutations in genes encoding sarcomeric proteins such as MYH7 and MYBPC3. The hypertrophy leads to diastolic dysfunction, myocardial ischemia, and increased LV filling pressures. HCM is associated with a high risk of arrhythmias, including AF and VT, due to myocardial disarray and fibrosis. The arrhythmogenic substrate is commonly localized intramurally within the hypertrophied segments of the septum and LV apex, though in cases with apical aneurysms, it may also involve the apical region [5].

### 2.3. Arrhythmogenic Right Ventricular Cardiomyopathy (ARVC)

ARVC is a myocardial disease that affects the right ventricle (RV), the LV or both, defined by fibrofatty myocardial replacement that causes systolic ventricular dysfunction and potentially lethal VAs that may lead to SCD especially in athletes and young people. Affected individuals usually manifest symptoms between the second and fourth decade of life, with about 60% of them showing pathogenic mutations in the genes encoding the cardiac desmosome, such as plakophilin-2 and desmoglein-2. While ARVC is commonly associated with the RV, there is a growing acknowledgment of its early and/or predominant impact on the LV. The criteria for the diagnosis of ARVC were first established in 1994 and later revised in 2010, when a Task Force found that the existing criteria showed a relatively good accuracy for the diagnosis of the right ventricular form, but a lower sensitivity for the diagnosis of the left-sided phenotypes. The 2020 Padua criteria have included the use of cardiac magnetic resonance (CMR) to noninvasively detect late-gadolinium enhancement (LGE) and/or myocardial fibrosis which are essential for the characterization of both the biventricular and left variants [6,7,8,9,10].

### 2.4. Non-Dilated Left Ventricular Cardiomyopathy

Non-dilated left ventricular cardiomyopathy is a term used to encompass all cardiomyopathies presenting with LV involvement without a dilative phenotype and without enough diagnostic criteria to be included in the other cardiomyopathy subtypes. This category may include ARVC with isolated LV involvement without a dilative phenotype, left ventricular non-compaction (LVNC) and Chagas disease, among others [11].

#### 2.4.1. Left Ventricular Non-Compaction (LVNC)

Currently not accepted as an individual entity, LVNC may be an adaptative phenotype to increased LV workload that can be encountered in normal (athletes) and pathological (DCM, valvular heart disease, etc.) conditions. LVNC is characterized by prominent trabeculations and deep intertrabecular recesses in the LV, resulting from arrested myocardial compaction during embryogenesis. The recesses pose significant challenges for procedural approaches, as they complicate precise targeting of ablation areas, thereby making catheter navigation considerably more difficult compared to cases without hypertrabeculation. This highlights the importance of addressing LVNC in discussions of VT ablation in the non-ischemic population.

With a prevalence estimated at 0.05% in the general population, LVNC can be sporadic or familial, with genetic mutations identified in sarcomeric and mitochondrial genes. The condition leads to systolic and diastolic dysfunction and is associated with arrhythmias, including VT and AF, due to the abnormal myocardial architecture. Arrhythmogenic regions are often localized to trabeculated and non-compacted segments of the LV [12].

#### 2.4.2. Chagas Disease

Chagas Disease is a parasitic infection caused by *Trypanosoma cruzi*, and it represents a significant cause of NICM, particularly in endemic regions. Myocardial fibrosis in Chagas cardiomyopathy typically involves the subepicardial or transmural layers, with patchy fibrosis areas and a predilection for the inferoseptal, inferior, lateral and posterolateral walls, which serve as common VT exit points. There is no specific LGE pattern, as the scar can be subendocardial, transmural (most common), subepicardial or localized within the midwall. The arrhythmogenic burden is further amplified by the presence of inflammation and autonomic dysfunction, hallmark features of the disease. These arrhythmias are frequently life-threatening, making arrhythmia management a central component of care in patients with advanced Chagas cardiomyopathy [13].

### 2.5. Restrictive Cardiomyopathy (RCM)

RCM is characterized by impaired ventricular filling with normal or near-normal systolic function and wall thickness. It is the least common form of NICM, accounting for less than 5% of cases. RCM can be idiopathic or associated with systemic diseases like amyloidosis. Genetic mutations have been identified in some familial cases. The restrictive filling pattern leads to elevated ventricular pressures and atrial enlargement. Arrhythmias, particularly AF and less commonly VT, are frequent due to atrial dilation and fibrosis. VT in RCM often arises in regions of atrial or ventricular fibrosis [14].

#### Cardiac Amyloidosis

Infiltration of the myocardium by amyloid fibrils leads to restrictive cardiomyopathy. The prevalence is increasing with improved diagnostic techniques. It can be acquired or hereditary, with mutations in the transthyretin gene in familial cases. Amyloid deposition leads to stiffening of the ventricular walls and impaired diastolic function. Arrhythmias, including AF and VT, are common due to infiltration and fibrosis of the conduction system. Arrhythmogenic foci often involve the atrioventricular node and ventricular myocardium [15].

## 3. Prevalence of Ventricular Tachycardia in NICM Subtypes

Ventricular tachycardia prevalence varies among NICM subtypes. DCM is the most frequently associated with VT, reflecting its high prevalence and significant myocardial scarring. Electrical storms are more frequently observed in DCM, a phenomenon that may not be solely attributed to extensive fibrosis and electrical remodeling associated with LV enlargement. Instead, it is primarily explained by DCM’s status as the most prevalent phenotype of NICM, encompassing a wide range of distinct genetic subtypes under a unified classification [16]. HCM and ARVC patients exhibit a high risk for VAs due to their underlying structural abnormalities and genetic predispositions, while RCM and LVNC patients exhibit lower but significant VT prevalence linked to specific fibrotic or architectural abnormalities.

Across this population, stratification of arrhythmic risk is crucial to guide decisions regarding ICD implantation, which remains a cornerstone of primary prevention of SCD. Unlike ischemic cardiomyopathy (ICM), where the risk stratification is more straightforward and typically tied to left ventricular ejection fraction (LVEF), NICM presents a much more complex scenario due to its heterogeneous substrates and variable disease evolution. Current risk scores, such as the HCM Risk-SCD score for HCM [17], integrate parameters like age, family history of SCD, unexplained syncope, and ventricular hypertrophy, yet its overall accuracy is far from what experts would prefer to use in clinical practice, and analogous tools for other NICM phenotypes remain less robust. It is worth mentioning, however, that despite clearer indications for ICM, SCD can occur in these patients even in the presence of an LVEF higher than 35%, despite the lack of formal ICD indications, underscoring the challenges of risk stratification. In NICM, the diversity of arrhythmogenic substrates, genetic variants, family history of SCD, and dynamic disease progression further complicates risk prediction. The electrophysiological (EP) study serves as a critical tool to identify high-risk individuals by unmasking electrical instability through inducible VAs, providing an individualized approach to risk assessment that complements clinical and imaging data. However, the final decision of whether or not to implant an ICD can ultimately be very challenging, particularly in those cases where the EP study remains negative despite an extensive potentially arrhythmogenic substrate [18].

Fibrosis, a hallmark of many NICM subtypes, creates the substrate for re-entry by disrupting normal electrical conduction. This can lead to slow conduction pathways and functional conduction blocks that facilitate re-entrant circuits. The distribution and extent of fibrosis vary across subtypes; for instance, epicardial or mid-myocardial fibrosis is common in ARVC and DCM, while diffuse fibrosis is often observed in cardiac amyloidosis and HCM [5,15].

In addition to structural abnormalities, ion channel dysfunction and genetic mutations also play pivotal roles in VT mechanisms. Conditions such as laminopathies and SCN5A mutations alter the expression or function of cardiac ion channels, predisposing patients to abnormal automaticity or triggered activity due to afterdepolarizations [2,3]. These electrophysiological changes are further exacerbated by autonomic imbalances, which can modulate conduction properties and trigger arrhythmias.

The most prevalent subtypes of NICMs, their fibrosis distribution and the most common VT exit points that they are associated with are described in Table 1.

**Figure 1:**
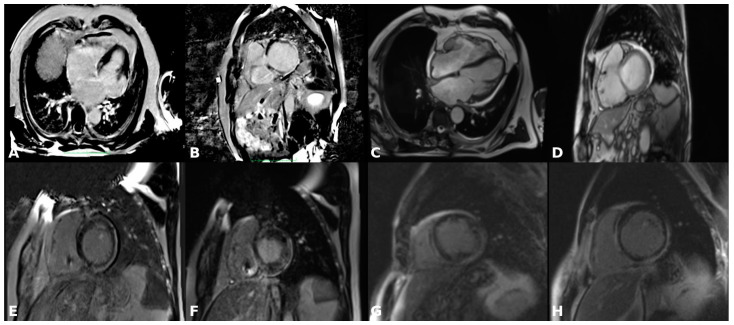
Fibrosis distribution patterns across various subtypes of NICM (all images belong to the authors’ personal imaging data collection and have been fully anonymized beforehand). (**A**,**B**) Anterolateral and inferolateral transmural scar in a patient with postmyocarditis dilated cardiomyopathy. (**C**,**D**) Diffuse scar in an idiopathic dilated cardiomyopathy. (**E**,**F**) “Ring-like” subepicardial fibrosis distribution seen on the anterior, anterolateral, inferolateral and inferior wall in a patient with ARVC with a desmoplakin mutation and biventricular involvement. (**G**,**H**) Late gadolinium enhancement with mid-myocardial distribution in the mid-lateral segments, as well as in the interventricular septum in a patient with LVNC.

Figure 1 illustrates examples of fibrosis distribution patterns across various subtypes of NICM.

## 4. Pre-Procedural Imaging: VT Substrate Assessment Through Multimodality Imaging Techniques

Pre-procedural cardiovascular multimodality imaging is crucial for successful VT ablation, as it provides valuable insights into each patient’s unique profile. These imaging techniques help clinicians understand the arrhythmogenic substrate and plan the most appropriate ablation approach. While each modality has its own strengths and limitations, they offer complementary information to guide effective treatment.

### 4.1. Late Gadolinium Enhancement Magnetic Resonance Imaging (LGE-MRI)

LGE-MRI is the gold standard for detecting myocardial fibrosis, providing high-resolution images of scar tissue. It is particularly valuable for identifying VT substrates in DCM, HCM, and ARVC, where fibrosis correlates with arrhythmic risk. The ability to delineate scar borders facilitates precise ablation targeting [19].

### 4.2. Multi-Detector Computed Tomography (MDCT)

Multidetector computed tomography (MDCT) imaging provides better anatomical resolution, making it particularly valuable in the planning of epicardial ablation procedures. Its utility is highlighted in identifying fat infiltration in ARVC and facilitating precise catheter placement in complex cases. MDCT is an excellent alternative for patients unable to undergo magnetic resonance imaging (MRI) due to contraindications, such as non-MRI-compatible implantable cardiac devices, foreign materials, severe anxiety, or significant imaging artifacts from existing devices that render MRI unsuitable for preprocedural planning. Additionally, MDCT offers critical insights into myocardial wall thickness, a parameter increasingly recognized for its ability to identify fibrotic areas, as regions of reduced thickness often correlate with myocardial scarring. The technique also boasts practical advantages, including faster acquisition times, reduced reliance on respiratory gating compared to MRI, lower cost, and broader availability. However, these benefits are counterbalanced by the inherent disadvantage of ionizing radiation exposure [20].

### 4.3. Echocardiography

Echocardiography, while limited in its ability to characterize tissue, remains a cornerstone for evaluating ventricular function and chamber dimensions. However, LGE-MRI is considered the gold standard for this purpose. Advanced echocardiographic techniques, such as strain imaging and three-dimensional reconstructions, offer the ability to detect subtle myocardial dysfunction that may not be apparent using traditional parameters, such as LVEF. LVEF, while widely validated, can be influenced by various conditions and may remain within normal limits despite the presence of subclinical structural heart disease (SHD). This is particularly relevant in NICM, where the pathological substrate is less apparent than in advanced coronary artery disease, especially during the early stages. Although echocardiography is less accurate than other imaging modalities for quantifying chamber volumes and sizes, it plays a pivotal role in the diagnosis and management of NICM due to its superior temporal resolution, cost-effectiveness, ease of use, and widespread availability in clinical practice.

### 4.4. Positron Emission Tomography (PET)

PET imaging identifies active inflammation and metabolic abnormalities, aiding in diagnosing inflammatory cardiomyopathies like sarcoidosis, which may necessitate tailored VT management strategies. Additionally, myocardial beta-adrenergic receptor density, assessed through PET, is lower in ARVC patients compared to age-matched controls, which might be explained by the altered presynaptic catecholamine reuptake or by elevated activity levels of the efferent neurons, ultimately leading to elevated levels of norepinephrine at the local synaptic site. Despite the potential of nuclear imaging modalities for risk assessment in other NICM subtypes, further research is needed [20].

## 5. Ablative Techniques

The field of catheter ablation has undergone significant advancements to address the complex challenges posed by VAs in patients with NICM. These innovations have expanded the arsenal of techniques available to electrophysiologists, each tailored to overcome the unique arrhythmogenic substrates and characteristics of NICM [21].

### 5.1. Endocardial Ablation

Endocardial ablation is the cornerstone of catheter-based therapies for ventricular arrhythmias and typically serves as the initial approach. This technique targets re-entrant circuits or focal triggers located endocardially, and is particularly effective for subendocardial substrates, which are common in ischemic cardiomyopathies but may also occur in a significant number of NICM patients. While highly effective in many cases, endocardial ablation can be of limited utility when the arrhythmogenic substrate is intramural or epicardial, requiring alternative strategies [21].

### 5.2. Epicardial Ablation

Epicardial ablation is particularly beneficial in conditions such as ARVC, Chagas disease, and select subtypes of DCM, where arrhythmogenic tissue is predominantly localized in the epicardium or subepicardium. Although this approach is more invasive than endocardial ablation and involves pericardial access—which may pose additional risks depending on patient-specific factors such as prior cardiac interventions and the need for an epicardial surgical window—it significantly enhances procedural success in cases where endocardial ablation alone proves inadequate. Potential complications of epicardial ablation include injury to adjacent structures such as the coronary arteries and phrenic nerve, as well as the risk of pericarditis, cardiac tamponade, or, in rare instances, cardiogenic shock [21].

### 5.3. Bipolar Ablation

Bipolar radiofrequency catheter ablation (Bi-RFCA) is an emerging ablation technique in which alternating current is delivered between two ablation catheters positioned on opposite sides of the target myocardium. One catheter is connected to the active port of the RF generator (active catheter, AC), while the other is connected to the indifferent port, replacing the conventional ground patch (return catheter, RC). Clinical case reports and series have demonstrated Bi-RFCA’s efficacy in managing VAs resistant to standard unipolar RFCA (Uni-RFCA) [22,23,24].

A recent retrospective registry, the largest published on bipolar ablation to date, reported a relatively high success rate, with 61% of surviving VT patients remaining VT-free during follow-up. This evidence suggests that Bi-RFCA could serve as a viable alternative for patients with recurrent VT following unipolar RFCA, particularly those with intramural substrates or arrhythmias originating from the LV summit [25]. The LV summit poses unique challenges due to its anatomical proximity to the left anterior descending and left circumflex coronary arteries, the presence of thick epicardial fat layers, and fibrotic components of the aortic and pulmonic valves. Additionally, the substantial thickness of regions such as the interventricular septum (IVS) complicates ablation further [26,27,28,29].

Structural heart disease often involves scar and adipose tissue, which hinder RF energy penetration and limit lesion depth. The variable resistivity of scarred tissue leads to uneven energy absorption and non-uniform tissue injury, complicating lesion formation with Uni-RFCA. In such scenarios, Bi-RFCA offers advantages by delivering energy more effectively to intramural substrates. Studies have shown that while higher-power Uni-RFCA may improve outcomes, it is associated with increased complication and mortality rates [30,31,32,33]. Conversely, Bi-RFCA achieves effective lesion formation with lower power, reflecting its greater efficiency as demonstrated in preclinical and clinical studies [25,34].

Another anatomical challenge addressed by Bi-RFCA is the heat sink effect, wherein blood flow in major vessels like the aorta or coronary arteries rapidly dissipates thermal energy, preventing effective lesion formation during RF application. Uni-RFCA is particularly susceptible to this phenomenon, leading to suboptimal results in scar-related VT. Bi-RFCA mitigates this effect by concentrating energy delivery between bipolar electrodes, enhancing lesion quality and depth [35].

The effectiveness of Bi-RFCA can also be influenced by electrode characteristics. Studies have highlighted the benefits of using 8 mm catheters in a parallel orientation for optimizing Bi-RFCA lesions, particularly in the great cardiac vein (GCV). Larger-tip RCs positioned in the venous system or on the right ventricular side of the IVS are advantageous for ablations targeting the LV summit or intramural substrates, as they reduce overheating risks and improve energy distribution [36,37,38].

Intramural VAs often exhibit multiple early activation sites, complicating precise endocardial mapping and requiring sequential ablation for durable suppression. Targeting only a single activation site often results in incomplete arrhythmia control due to the deep intramural origin of these arrhythmias. By altering the vector of RF current flow, Bi-RFCA extends lesion dimensions, improving outcomes. Various electrode configurations may be employed to maximize ablation efficacy, especially in cases refractory to unipolar approaches [25].

Although Bi-RFCA shows high acute efficacy and acceptable safety in most VT patients refractory to Uni-RFCA, current studies have limitations. These include small patient populations, inconsistent ablation settings, and short follow-up periods. Additionally, the criteria for selecting bipolar ablation and its optimal parameters remain inadequately defined. Future research should prioritize validating these findings through larger, prospective, controlled trials and directly comparing Bi-RFCA with other innovative ablation techniques.

### 5.4. Half-Saline-Infused Ablation

Conventional open-irrigated catheters typically use a 0.9% saline solution as the irrigant. In contrast, hypotonic 0.45% saline (half-normal saline—HNS) has a higher impedance, approximately 180 Ohm compared to 90 Ohm for normal saline (NS). When used as the irrigation fluid, this increased impedance around the ablation tip reduces the dissipation of electrical current into the blood pool, thereby concentrating RF current delivery into the myocardial tissue. Studies conducted both ex vivo and in vivo have demonstrated that this approach can produce deeper ablation lesions at the same power settings in high-flow open-irrigated catheters [39].

A study involving 935 consecutive patients undergoing VT RFCA, which included 900 procedures using HNS guided by impedance drop and assisted by intracardiac echocardiography (ICE), demonstrated a high acute success rate and an acceptable complication rate, with 13% of patients experiencing adverse effects within the first 30 days post-procedure [40].

The role of HNS RFCA has been further explored in various studies, evaluating its use either as a primary therapeutic approach or as an alternative strategy in patients with recurrent VTs where NS ablation was unsuccessful. Some evidence indicates that HNS ablation achieves higher acute success rates; however, it has also been associated with increased VT recurrence during follow-up and greater radiation exposure. Strategies to mitigate radiation exposure, such as enhanced operator experience to reduce procedure time or the use of ICE, could address these concerns. Nevertheless, the potential for different outcomes when using HNS as the initial irrigation agent requires further investigation [41].

Conversely, the HALF study, a randomized trial with patients equally assigned to HNS or NS ablation groups with comparable baseline characteristics, found that HNS ablation achieved similar success and safety outcomes to NS ablation. Notably, HNS procedures were associated with shorter ablation times, suggesting a potential procedural efficiency advantage [42].

For epicardial ablation, it has been proposed that HNS could theoretically help safeguard the parietal pericardium from heat-related damage, provided that the catheter is properly oriented toward the epicardial surface [39].

These findings highlight the need for additional research to clarify the optimal clinical contexts for HNS use and to further assess its long-term efficacy and safety.

### 5.5. Surgical Ablation

In recent years, significant advancements have been made regarding various RFCA techniques for patients with VAs. However, certain circuits—particularly those unrelated to myocardial scarring—remain challenging to address using conventional techniques. For individuals with circuits that are inaccessible, contraindications to percutaneous epicardial access, or who have previously undergone unsuccessful catheter ablation procedures, surgical ablation may serve as a viable alternative.

Anter et al. assessed eight patients with NICM and refractory VTs despite receiving optimal antiarrhythmic therapy. These patients had undergone unsuccessful catheter ablation procedures, either endocardial or epicardial. Surgical cryoablation was performed at sites identified using three-dimensional electro-anatomical mapping (3D EAM) acquired during the percutaneous procedure. Over an average follow-up period of 23 ± 6 months, interrogation of ICDs demonstrated a notable reduction in VT burden, proven by fewer appropriately delivered internal shocks [43].

Similarly, a study conducted by Kunkel et al. included eight consecutive patients experiencing refractory electrical storm and contraindications to transcutaneous epicardial access. These individuals underwent surgical ablation guided by intraoperative electro-anatomical landmarks. All patients achieved clinical non-inducibility of VT, and during a mean follow-up of 3.4 ± 1.7 years, VT burden significantly decreased, with 75% remaining free of electrical storms [44].

Surgical ablation may be a viable option for VT patients undergoing left ventricular assist device (LVAD) implantation, especially those with pericardial adhesions from prior surgeries that complicate epicardial mapping and ablation. Given their higher procedural risks and mortality, enhanced precautions are essential. Combining surgical VT ablation with LVAD implantation may effectively address epicardial VT substrates and reduce post-implantation VT burden [45].

Although surgical VT ablation is not considered the gold standard, its integration with 3D EAM represents a dependable option for patients with recurrent, treatment-resistant VT, those contraindicated for standard approaches, those with VT arising from anatomically challenging or inaccessible areas, or those requiring concurrent open-heart surgical interventions.

### 5.6. Needle Ablation

Certain patients may present with a VT substrate primarily located on the epicardial surface, embedded deep within the myocardium, or undetectable using multimodality imaging or 3D EAM. Despite advancements in standard techniques, endocardial RF lesions may sometimes fail to achieve sufficient size or depth to disrupt the arrhythmic circuit. In cases where an epicardial substrate is suspected, epicardial mapping and ablation can be pursued, provided the operator has the required expertise and is mindful of associated risks, such as perforation and tamponade. However, this is often limited by external factors such as thick layers of epicardial fat, proximity to critical structures, and substrates located too deep within the myocardium to be accessible via the standard approaches [46].

Intramyocardial needle ablation, developed over the past two decades, involves delivering saline into the myocardium via a retractable needle electrode, enabling deeper and larger lesions with improved energy delivery.

Preclinical and clinical studies have demonstrated its promise as a salvage strategy for patients with recurrent or refractory VT after failed catheter ablations. By providing targeted ablation at virtually any site and producing larger lesions while maintaining a reasonable safety profile, intramyocardial needle ablation is emerging as a valuable alternative for complex VT cases, with ongoing improvements expected as global expertise expands [47,48,49].

### 5.7. Transarterial Coronary Ethanol Ablation and Retrograde Coronary Venous Ethanol Ablation

TCEA (Transarterial Coronary Ethanol Ablation) and RCVEA (Retrograde Coronary Venous Ethanol Ablation) are alternative methods developed for patients with arrhythmogenic myocardial substrate located deep in the myocardium, where standard techniques may not reach, while aiming to protect surrounding structures.

TCEA was first described over 30 years ago in canine models [50] and later applied clinically by Brugada et al. in patients with ischemic VTs, yielding promising results [51,52] that were soon supported by other reports. The procedure involves identifying the coronary artery supplying the arrhythmogenic tissue and selectively cannulating it with an angioplasty balloon. Once the correct artery is accessed, the balloon allows targeted delivery of ethanol. Follow-up studies have reported noninducibility rates ranging from 56% to 84%. However, TCEA is currently viewed as a less consistent alternative to conventional ablation techniques [53].

RCVEA, in contrast, offers certain advantages. It delivers ethanol through the venous system, which allows for a higher ethanol dilution due to retrograde flow, reduces the risk of myocardial injury, and avoids arterial cannulation, thus lowering procedural complexity and related complications [53].

The RCVEA procedure begins with 3D EAM to pinpoint the earliest endocardial VT activation site. The coronary sinus (CS) is cannulated, and the GCV and anterior interventricular vein (AIV) are examined with venograms to identify the branches closest to the target site. A guidewire is advanced into the vein, providing support for a preloaded angioplasty balloon. Once the positioning is verified, the guidewire is removed, the balloon is deployed, and contrast is injected to confirm myocardial staining—an indirect marker of accurate targeting. Ethanol, typically at a concentration of 96–98%, is administered incrementally until a therapeutic reaction, such as increased echogenicity on intracardiac echocardiograms, is observed [53].

In cases with atypical venous anatomy, challenges can arise. However, strategies such as the “double-balloon technique” can address these issues. This method uses a secondary balloon to block collateral blood flow, enabling effective ethanol delivery to remote or extensive areas [54].

TCEA and RCVEA expand therapeutic options for patients with refractory VT, offering feasible alternatives in complex cases where standard techniques fail to meet expectations.

### 5.8. Ultra-Low-Temperature Cryoablation

Over the past decade, the frequency of VT ablations has risen steadily, accompanied by the development of innovative techniques as the field has advanced. However, there is growing recognition that conventional irrigated-tip RF ablation catheters may not be the optimal tools for VT ablation. The depth of RF-induced lesions is often restricted to 3–5 mm due to the presence of scar tissue, while the ventricular wall thickness typically ranges from 6 to 12 mm. This limitation reduces the efficacy of RF ablation beyond the immediate endocardial and sub-endocardial layers, necessitating the incorporation of adjunctive approaches such as epicardial ablation and complex multi-catheter techniques. Ultra-low-temperature cryoablation (ULTC) presents a promising alternative to conventional RF ablation for VT. This technique utilizes a “near-critical” nitrogen refrigerant near its boiling point of −196 °C, which exhibits the flow properties of a gas combined with the density and thermal capacity of a liquid. This allows continuous delivery through catheters with narrow lumens. In preclinical studies, ULTC has been shown to create lesions with adjustable depths ranging from 4 mm to over 10 mm, effectively penetrating chronic scar tissue, which potentially positions ULTC as a superior technique for these procedures [55].

A recently published first-in-human study (Cryocure-VT) evaluating ULTC in patients with SHD due to ischemic and non-ischemic etiologies demonstrated encouraging outcomes. At the six-month follow-up, 81% of patients remained free from ICD therapies, and 60.3% were free of VT recurrences, with comparable efficacy observed between ischemic and non-ischemic cohorts. However, it is noteworthy that patients with NICM accounted for only 22.3% of the study population, and all procedures were limited to endocardial ablation. These factors constrain the generalizability of the findings, particularly for NICM patients, where the arrhythmogenic substrate is frequently mid-myocardial or epicardial, potentially requiring adjunctive approaches such as bipolar or epicardial ablation. Importantly, the study reported a complication rate of 6%, consistent with rates observed in established VT ablation techniques, which underscores a favorable safety profile and highlights the potential of ULTC as an effective and safe modality for VT ablation [55].

A clinical trial investigating ULTC in patients with scar-mediated ischemic and non-ischemic sustained monomorphic VTs (FULCRUM-VT, NCT05675865) is currently underway. This trial aims to play a pivotal role in evaluating the efficacy and safety of this technique, with preliminary results anticipated in the near future [56].

Additionally, the combination of PFA with ULTC holds promise for generating more effective transmural lesions compared to either modality alone. This concept has been previously explored [57], and recent preclinical studies in swine models demonstrated that pulsed field cryoablation (PFCA) induces deeper lesions while reducing muscle contractions and microbubble formation. However, these studies have thus far been limited to atrial myocardium. A notable limitation of PFA is its restricted lesion depth, with most current systems optimized for left atrial applications, achieving penetration of only 2–5 mm. For thicker myocardial regions, such as parts of the left atrium or ventricular tissue, greater lesion depths are essential. While certain parameters (such as voltage, pulse duration, the number of pulses per train, the number of pulse trains, and the asymmetry of the biphasic waveform) can be adjusted to increase lesion depth, such modifications are, at least for the moment, associated with a heightened risk of complications [58].

Further studies on larger populations, including randomized trials, are necessary in order to validate these findings.

### 5.9. Remote Magnetic Navigation

The increased radiation exposure associated with VT RFCAs has driven the adoption of non-fluoroscopic techniques. Remote magnetic navigation (RMN) utilizes externally controlled magnets to generate a magnetic field, enabling precise guidance and positioning of catheters. This approach offers several advantages, including enhanced catheter control, significant reductions in radiation exposure, and a potentially lower risk of complications such as cardiac perforation and tamponade, attributable to the use of softer and more flexible catheter tips. Furthermore, improved catheter tip contact achieved with RMN facilitates the creation of larger and more homogeneous ablation lesions.

Multiple studies have evaluated the safety and efficacy of RMN, demonstrating reduced fluoroscopy and ablation times, as well as a decreased recurrence rate of VT. These findings suggest that RMN represents a promising advancement in catheter ablation techniques, particularly for complex arrhythmias requiring precise and effective interventions [59,60].

### 5.10. Novel and Emerging Approaches

In addition to these established techniques, novel approaches are being explored to further expand therapeutic options for VAs in NICM.

#### 5.10.1. Stereotactic Body Radiation Therapy (SBRT)

Stereotactic body radiation therapy (SBRT) represents a transformative approach in the management of ventricular tachycardia (VT), particularly for patients with refractory arrhythmias who are not candidates for traditional invasive procedures. This non-invasive technique delivers highly focused, high-dose radiation to arrhythmogenic myocardial substrates with millimeter precision, effectively altering the electrophysiological properties of the target tissue [61]. SBRT leverages imaging modalities such as cardiac CT, MRI, and electroanatomic mapping to accurately localize the arrhythmogenic regions [62].

Clinical studies have demonstrated the efficacy of SBRT in significantly reducing VT episodes and ICD shocks, with some patients achieving complete arrhythmia suppression [63,64]. Moreover, the procedure is performed as an outpatient therapy, minimizing hospital stays and associated costs. Despite its promise, SBRT effects on arrhythmia exhibit latency and the radiation exposure can be associated with potential risks, including off-target radiation effects on surrounding structures such as the esophagus or coronary arteries and long-term concerns about radiation-induced fibrosis [65]. Ongoing research, including randomized controlled trials, is required to refine patient selection, optimize dosing protocols, and evaluate long-term safety and efficacy.

#### 5.10.2. Neuraxial Modulation

Neuraxial modulation represents a novel approach to managing VAs by targeting the autonomic nervous system’s role in arrhythmogenesis. The technique modulates sympathetic and parasympathetic inputs to the heart, which are often heightened in patients with arrhythmia-prone substrates. Neuraxial modulation encompasses interventions such as thoracic epidural anesthesia, stellate ganglion block, or bilateral cardiac sympathetic denervation. These procedures aim to reduce the autonomic drive that triggers or sustains ventricular arrhythmias [66].

Studies have demonstrated the efficacy of neuraxial modulation in decreasing the burden of VAs, particularly in patients with VT storms or those unresponsive to antiarrhythmic medications and catheter ablation [67,68]. The intervention is especially beneficial in acute settings, providing rapid suppression of arrhythmias in critically ill patients. However, the procedure requires skilled operators and carries potential risks such as transient hypotension, local infection, or nerve damage [69]. Despite these challenges, neuraxial modulation is a promising adjunctive therapy for VT, particularly in cases driven by heightened autonomic tone or recurrent arrhythmias refractory to standard treatments. Further investigation is needed to define its long-term benefits and integrate it into broader clinical practice [70].

#### 5.10.3. Pulsed Field Ablation

Pulsed field ablation (PFA) is an innovative nonthermal ablation technique that employs ultrashort, high-voltage electrical pulses to induce microscopic pores in cell membranes, resulting in apoptotic cell death [24]. While already established as a treatment modality for AF, there is growing interest in its application for VAs [71,72,73]. Preclinical studies have shown that PFA can create effective transmural lesions in ventricular tissue, with lesion depths reaching up to 9.4 mm in swine models [74]. The limited clinical experience with PFA in real-world VT patients suggests that, despite certain advantages—including its ability to effectively target areas with poor catheter stability due to its reliance on proximity rather than direct contact, its rapid application, and its capacity to create larger lesions within a shorter timeframe, making it particularly useful for patients unable to hemodynamically tolerate inducible VTs during the procedure—the depth of lesions achieved in humans may fall short, as the only substrate that was completely eliminated was the endocardial one. This limitation, contrary to findings in swine models, raises concerns about its effectiveness in treating VTs originating from epicardial sites, even in the right ventricle [73].

A recent systematic review evaluating all relevant publications on PFA in patients with VTs reported an overall procedural success rate of 87.5%, with no procedural complications documented in the included studies. This encouraging success rate highlights the potential of PFA as a promising alternative to conventional ablation techniques. Nevertheless, it is important to recognize the limitations of the available studies, such as their small sample sizes and limited follow-up periods [75]. Further research is ongoing to assess the long-term safety and efficacy of this emerging technology.

These evolving techniques, while promising, require further validation through large-scale clinical trials to establish their long-term efficacy and safety. Together, they reflect the dynamic nature of the field, underscoring a commitment to addressing the diverse and challenging arrhythmogenic substrates encountered in NICM.

A detailed summary of various VT ablation techniques, including their definitions, relevant clinical trials, advantages, disadvantages, and limitations is presented in Table 2.

**Figure 2:**
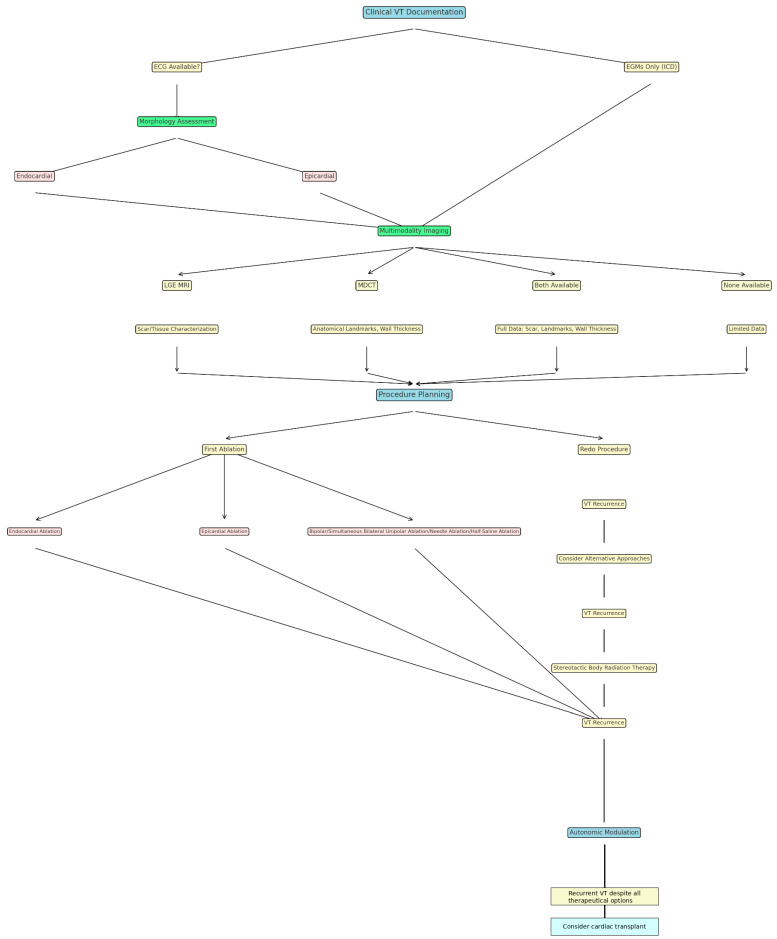
A schematic description of a step-by-step algorithm for the management of non-ischemic patients presenting with VT.

A more schematic step-by-step approach regarding the clinical workflow in the management of a non-ischemic patient presenting with VT is shown in Figure 2.

## 6. Future Perspectives

Emerging technologies are paving the way for a transformative era in the management of VAs, particularly in patients with NICM. High-resolution electroanatomic mapping systems are at the forefront, enabling clinicians to obtain detailed and precise representations of arrhythmogenic substrates. These systems improve the ability to delineate low-voltage areas, detect conduction abnormalities, and differentiate active arrhythmogenic sites from passive scar tissue. By enhancing substrate identification, these tools are expected to optimize ablation strategies, reduce procedural times, and improve success rates [81,82].

Machine learning algorithms further complement these advances by leveraging large datasets to refine procedural planning and outcome prediction. These algorithms analyze complex patterns in electrocardiograms, imaging studies, and clinical data to identify predictors of VT recurrence or ablation failure [83,84]. Moreover, artificial intelligence (AI) holds potential in personalizing treatment approaches by integrating genetic, phenotypic, and electrophysiological characteristics to tailor interventions to individual patients [85].

Genetic therapies targeting underlying mutations represent another frontier in VA management. Advances in gene editing technologies, such as CRISPR/Cas9, offer the possibility of correcting pathogenic mutations in inherited arrhythmogenic conditions like ARVC or laminopathies, potentially reducing or even eliminating the need for invasive procedures [86,87].

In recent years, notable advancements have been made in the treatment of ARVC, particularly in exploring recombinant adeno-associated virus (AAV)-mediated gene therapy [88]. This approach has shown potential in preclinical models to slow the progression of plakophilin-2 (PKP2)-associated ARVC and improve cardiac function [89,90,91,92]. Currently, three AAV-mediated gene therapies targeting PKP2-associated ARVC have received FDA approval to proceed to phase 1 clinical trials. These therapies are designed for patients with confirmed pathogenic PKP2 variants identified through genetic testing and a high frequency of premature ventricular complexes [88].

These developments mark a significant step forward in establishing AAV-mediated gene therapy as a promising treatment option for cardiac genetic disorders, offering new hope for conditions previously considered difficult to manage.

Non-invasive approaches, particularly SBRT, offer exciting alternatives for patients who are poor candidates for catheter ablation. By delivering precisely focused radiation to arrhythmogenic substrates, SBRT provides a non-invasive means to modify arrhythmogenic tissue. Early clinical studies have demonstrated promising results, with reductions in VT burden and improvements in patient outcomes [59,60]. Ongoing research aims to refine dosing protocols, improve targeting accuracy, and minimize potential long-term radiation-related risks.

To ensure these innovations transition effectively from experimental settings to routine clinical practice, collaborative research efforts are paramount. Multicenter clinical trials will play a crucial role in validating the safety, efficacy, and cost-effectiveness of these novel technologies. Such trials will also help standardize protocols, facilitate regulatory approval, and promote equitable access to these advanced therapies.

As these technologies continue to evolve, their integration into clinical workflows will require interdisciplinary collaboration among electrophysiologists, geneticists, radiologists, and data scientists. However, the implementation of these novel technologies may face challenges such as data privacy concerns, the need for robust validation in diverse patient populations, and the adaptation of existing healthcare infrastructure. By embracing these innovations, the field of VT management has the potential to achieve more precise, personalized, and effective care for patients with NICM and other forms of arrhythmogenic heart disease.

## 7. Conclusions

The management of ventricular arrhythmias in NICM patients requires a nuanced approach that considers the heterogeneity of disease subtypes and arrhythmogenic mechanisms. Advances in imaging and ablation technologies have significantly improved therapeutic outcomes, but challenges remain. Future research should focus on refining diagnostic tools, optimizing ablation strategies, and integrating novel therapies to enhance patient care. Additionally, the integration of cutting-edge innovations like artificial intelligence, high-resolution mapping, and non-invasive modalities into clinical practice holds immense potential, but their widespread adoption will require addressing hurdles such as infrastructure limitations, cost, and data privacy concerns. By fostering interdisciplinary collaboration and prioritizing patient-centered approaches, the field can achieve transformative progress in reducing the burden of arrhythmias and improving outcomes for this complex patient population.

## Figures and Tables

**Table 1:** NICM subtypes, fibrosis distribution, and most common VT exit points.

NICM Subtype	Typical Fibrosis Distribution	Most Common VT Exit Points
Dilated Cardiomyopathy (DCM)	Two fibrosis distribution patterns: a septal one (usually with an extension towards the anterior and the inferior segments) and one involving the basal LV lateral wall. The septal pattern has an overall worse prognosis and oftentimes requires a more complex ablative approach (bilateral unipolar approach, bipolar ablation, needle techniques, saline infused ablation, transcoronary ethanol ablation and surgical ablation).	Interventricular septum and the basal LV lateral wall
SCN5A Mutations	Fibrosis in the conduction system and adjacent myocardial tissue.	His-Purkinje system, interventricular septum
Laminopathies	Mid-myocardial or subepicardial, often basal LV anterior wall and septum, but can sometimes be confined to the inferior wall or the subaortic mitral continuity.	Septal and basal LV anterior wall, basal inferior wall and the mitroaortic continuity
Left Ventricular Non-Compaction (LVNC)	Subendocardial fibrosis with trabeculated myocardial layers, particularly in the apex and mid-ventricle.	Apical and mid-ventricular regions
Hypertrophic Cardiomyopathy (HCM)	Patchy fibrosis, predominantly in hypertrophied regions, septal involvement common; apical in cases where apical aneurysm is associated.	Septal regions, left ventricular outflow tract (LVOT), LV apex if an apical aneurysm is present
Arrhythmogenic Right Ventricular Cardiomyopathy (ARVC)	Subepicardial, predominantly in the right ventricular free wall (especially in peritricuspidian inflow area but also the outflow tract).	Right ventricular free wall, subtricuspidian, outflow tract
Cardiac Sarcoidosis	Focal and patchy, frequently in the basal septum and lateral walls.	Basal septum, inferolateral wall
Cardiac Amyloidosis	Diffuse interstitial fibrosis, commonly affecting the entire myocardium with no specific predilection. The first affected area is the subendocardial area, but its substrate is more diffuse than seen in ischemic heart disease.	Diffuse VTs, multiple sites due to diffuse myocardial involvement, initially subendocardially
Chagas Disease	No specific LGE pattern—the scar can be subendocardial, transmural (most common), subepicardial or localized within the midwall. The scar is frequently localized in the interventricular septum, as well as the inferior, lateral and inferolateral LV walls.	Inferior interventricular septum, inferior, lateral and inferolateral LV walls
Myocarditis	Patchy and diffuse, often involving the lateral wall and septum.	Commonly involving the lateral wall and interventricular septum

**Table 2:** VT ablation techniques: definitions, clinical trials, pros, cons, and limitations.

Ablation Technique	Definition	Clinical Trials	Pros	Cons	Limitations	References
Endocardial Ablation	Ablation performed from the inner surface of the heart chambers.	VANISH Trial: Compared catheter ablation with escalated antiarrhythmic drug therapy in patients with ischemic cardiomyopathy and VT.	Minimally invasive; effective for subendocardial substrates.	Limited efficacy for epicardial or intramural substrates.	May not reach deeper or epicardial arrhythmogenic foci.	[76,77]
Epicardial Ablation	Ablation performed from the outer surface of the heart.	No specific large RCTs; observational studies suggest efficacy in non-ischemic cardiomyopathies.	Accesses arrhythmogenic substrates inaccessible endocardially.	Invasive; risk of complications like coronary artery or phrenic nerve injury.	Requires pericardial access; potential for procedural complications.	[77,78,79]
Bipolar Ablation	Ablation using two electrodes to create a circuit, allowing deeper lesion formation.	Limited clinical trial data; primarily case series and observational studies.	Creates deeper lesions with lower power; potentially more effective for intramural substrates.	Technically challenging; requires precise electrode positioning.	Limited availability; lack of standardized criteria for selecting the individuals suitable for bipolar ablation; requires further extensive clinical trial validation.	[25]
Half-Saline Infused Ablation	Ablation with half-normal saline irrigation to cool the electrode, allowing higher power delivery and deeper lesions.	Limited RCTs available (the HALF study); supported by small prospective studies and retrospective analyses.	Enables the creation of larger, deeper lesions. When the power is guided by impedance drop (and, if possible, assisted by ICE) it has a high acute success rate and a safety profile similar to NS ablation.	Risk of steam pops; potential for tissue overheating if not calibrated correctly.	Limited by catheter design and operator experience.	[40,41,42]
Surgical Ablation	Open-heart surgery to remove or isolate arrhythmogenic tissue.	Historical studies; largely supplanted by catheter-based techniques.	Direct visualization; effective for extensive substrates.	Highly invasive; significant morbidity and recovery time.	Reserved for patients undergoing cardiac surgery for other indications or those with refractory VAs and an extensive substrate inaccessible with standard approaches.	[43,44]
Needle Ablation	Percutaneous needle delivery of energy directly into myocardial tissue.	Emerging technique; limited clinical data available (the SERF VT study).	Targets deep intramural substrates; precise lesion placement.	Invasive; potential for myocardial perforation.	Experimental; not widely adopted in clinical practice.	[47,48,49]
Transarterial Coronary Ethanol Ablation (TCEA)	Injection of ethanol into coronary arteries supplying arrhythmogenic tissue to induce necrosis.	Limited to case reports and small series.	Effective for select cases with well-defined arterial supply to VT focus.	Risk of coronary artery damage; myocardial infarction.	Highly selective patient selection required; not widely practiced.	[80]
Retrograde Coronary Venous Ethanol Ablation (RCVEA)	Injection of ethanol into coronary veins to target arrhythmogenic tissue.	Sparse clinical data; limited to small case series.	Higher ethanol dilution due to retrograde flow, low risk of myocardial injury and potential arterial cannulation; lower complication rate than the transarterial approach.	Risk of venous thrombosis; limited efficacy data.	Experimental; lack of standardized protocols.	[53]
Ultra-Low-Temperature Cryoablation (ULTCA)	Near-critical nitrogen refrigerant near its boiling temperature of −196 °C continuously injected through small lumen catheters to penetrate deep scar areas and reach intramural substrates.	Limited clinical data; one clinical trial published to date (Cryocure-VT). One pivotal clinical trial currently under development (FULCRUM-VT).	Ability to create deeper lesions, reach intramural substrates and penetrate chronic scar tissue with potentially less complications than high-energy RF.	Requires specialized equipment; steep learning curve.	Early stages; published trials with short follow-up period; not widely adopted in clinical practice; limited by operator experience and lack of standardized protocols; requires further extensive clinical trial validation.	[55]
Remote Magnetic Navigation (RMN)	Use of magnetic fields to remotely steer ablation catheters within the heart.	Studies indicate safety and efficacy in various arrhythmias, including VT.	Enhanced catheter stability; reduced radiation exposure.	Requires specialized equipment; steep learning curve.	Limited availability; high procedural costs.	[59]
Pulsed Field Ablation (PFA)	Application of ultrashort, high-voltage electrical pulses to create microscopic pores in cell membranes and induce apoptosis.	Already an established therapy for AF; an emerging technique for VT. Limited to case reports and small series.	Able to target areas with poor catheter stability, fast applications with larger lesions in a shorter timeframe (may be useful in patients who cannot hemodynamically tolerate the inducible VTs).	Lesions may not be completely transmural; has not proven effective on epicardial substrate.	May not reach deeper or epicardial arrhythmogenic foci. Further research is required.	[73]

## Data Availability

Not applicable.
